# Genetic and environmental perturbations alter the rhythmic expression pattern of a circadian long non-coding RNA, *Per2AS*, in mouse liver

**DOI:** 10.12688/f1000research.125628.2

**Published:** 2022-10-31

**Authors:** Lin Miao, Kyle R. Batty, Ayana N. Jackson, Heather A. Pieno, Maisy W. Rhoades, Shihoko Kojima

**Affiliations:** 1 Department of Biological Sciences, Virginia Tech, Blacksburg, VA, 24061, USA; 2 Fralin Life Sciences Institute, Virginia Tech, Blacksburg, VA, 24061, USA; 3 Division of Systems Biology, Academy of Integrated Science, Virginia Tech, Blacksburg, VA, 24061, USA

**Keywords:** LncRNAs, circadian rhythm, antisense, rhythmicity, feeding regimen

## Abstract

**Background**: Long non-coding RNAs (lncRNAs) play a wide variety of biological roles without encoding a protein. Although the functions of many lncRNAs have been uncovered in recent years, the regulatory mechanism of lncRNA expression is still poorly understood despite that the expression patterns of lncRNAs are much more specific compared to mRNAs. Here, we investigated the rhythmic expression of *Per2AS*, a novel lncRNA that regulates circadian rhythms. Given that *Per2AS* expression is antiphasic to *Period2* ( *Per2*), a core circadian clock gene, and transcribed from the antisense strand of *Per2*, we hypothesized that the rhythmic *Per2AS* expression is driven either by its own promoter or by the rhythmic *Per2* transcription via transcriptional interference.

**Methods**: We leveraged existing circadian RNA-seq datasets and analyzed the expression patterns of *Per2AS* and *Per2* in response to the genetic or environmental disruption of the circadian rhythm in mouse liver. We tested our hypotheses by comparing the changes in the expression patterns of *Per2AS *and *Per2*.

**Conclusions**: We found that, in some cases, *Per2AS *expression is independently controlled by other circadian transcription factors. In other cases, the pattern of expression change is consistent with both transcriptional interference and independent regulation hypotheses. Although additional experiments will be necessary to distinguish these possibilities, findings from this work contribute to a deeper understanding of the mechanism of how the expression of lncRNA is regulated.

## Introduction

Long non-coding RNAs (lncRNAs) are a subgroup of RNAs longer than 200 nucleotides that do not produce proteins and play a variety of roles in a number of biological processes, including innate immune response, ^1^ cell cycle control, ^2^ cell differentiation, ^3^^,^^4^ X-inactivation, ^5^^,^^6^ and neuronal activity. ^7^^,^^8^ Because lncRNAs do not produce proteins, it is important for lncRNAs to interact with other molecules, such as other nucleic acids and RNA-binding proteins, to exert their functions. For example, nuclear lncRNAs interact with DNA, chromatin, and proteins and modulate nuclear processes, like chromatin organization, RNA transcription, splicing, and lncRNA nuclear export and retention. In contrast, cytoplasmic lncRNAs interact with proteins, other RNAs, or different organelles to alter mRNA stability and localization, protein translation, post-translational modification, mitochondrial functions, or protein trafficking. ^9^^–^^15^ In some cases, the act of transcription, rather than the lncRNA transcripts, is the functional entity to regulate target gene expression locally. ^16^

Although significant progress has been made in understanding the functions of lncRNAs in recent years, their transcription regulatory mechanism has been poorly understood. Similar to mRNAs which are transcribed by RNA Polymerase II, a considerable number of lncRNAs are also transcribed by RNA Polymerase II, 5′ capped, 3′ polyadenylated, and multi-exonic. ^17^ Interestingly, however, lncRNAs exhibit more cell type-, tissue-, developmental stage- or disease state-specific expression patterns compared to mRNAs. ^18^^–^^21^ This has raised many interesting questions: What are the regulatory mechanisms of lncRNA transcription to achieve highly specific expression patterns? Is the transcription of lncRNAs also regulated by transcription factors which bind to its promoter/enhancer, similar to mRNAs? If so, why is lncRNA expression more specific? Is there a universal mechanism to regulate the transcription of all lncRNAs, or is the transcription of different lncRNAs regulated by different mechanisms? Does the expression of lncRNAs respond to external inputs similar to mRNAs?

We have recently shown that *Per2AS*, a lncRNA, plays an important role in regulating circadian rhythms, ^22^ an internal timing mechanism to anticipate and respond to daily environmental rhythms driven by the rotation of the Earth. Interestingly, *Per2AS* is transcribed from the antisense strand of *Period 2* ( *Per2*), one of the core clock genes essential for generating circadian rhythmicity, and its expression is rhythmic and antiphasic to *Per2* mRNA. ^23^^–^^26^ Most strikingly, we further demonstrated that the transcription of *Per2AS*, rather than its transcripts, is important for regulating circadian rhythms. ^22^ These data prompted us to interrogate how rhythmic *Per2AS* transcription is regulated.

In this study, we analyzed the expression patterns of *Per2AS* under conditions in which the circadian clock was perturbed either genetically or environmentally. We used publicly available circadian transcriptomic datasets from mouse liver, where *Per2AS* is abundantly and rhythmically expressed. ^23^^–^^26^ Our first hypothesis is that the rhythmic *Per2AS* transcription is regulated by rhythmic antiphasic transcription of *Per2* by means of transcriptional interference, in which the transcription process on one strand suppresses the transcription process of the other strand. ^27^^–^^29^ Our alternative hypothesis is that rhythmic *Per2AS* expression is driven by its own promoter, similar to mRNAs. ^30^^–^^33^ If the former is true, we anticipate that changes in *Per2* and *Per2AS* expression would always be antiphasic. If the latter, then we anticipate that a change in *Per2AS* expression would be independent of that of *Per2.* We also take into account the possibility that the two hypotheses are not mutually exclusive. Results from this study contribute to our mechanistic understanding of how circadian rhythm is regulated by *Per2AS* and, more broadly, how the transcription of antisense lncRNAs is regulated.

## Methods

### RNA-seq data acquisition and processing

All the fastq files were obtained from NCBI SRA (GSE135898, GSE135875, GSE107787, GSE102072, GSE143528, ^34^^–^^37^ except for PRJDB7789, which was obtained from DDBJ DRA (PRJDB7789). ^38^ Fastq reads were mapped to the Ensembl mouse genome release 38 (mm10) using STAR 2.7.7a ^39^ with outFilterScoreMinOverLRead = 0.3 and outFilterMatchNMinOverLRead = 0.3 options. The ‘condenseGenes’ option was also used to select the most abundant isoform of each gene. The mapped reads were quantified by HOMER (v 4.11.1) ^40^ and normalized by the transcripts per million (TPM) option. We used the -sspe option for paired-end reads and the -strand – or -strand + option to quantify mapped reads in a strand-specific manner. The option -bp10 was used for GSE102072 and PRJDB7789 to filter alignments with mapQ smaller than 10. *Per2AS* expression was calculated from the antisense strand of the *Per2* genomic region as it is not annotated in Mus musculus GRCm38.95 GTF or NCBI RefSeq mm10 GTF files.

### Statistical analyses

We used the two-way analysis of variance (ANOVA) in Microsoft Excel to test for differences in RNA levels between the experimental groups (i.e., genotype, diet), except for the dataset GSE102072 in which some samples had only one biological replica. The rhythmicity of each RNA expression was assessed by MetaCycle, ^41^ which integrates three algorithms, ARSER, JTK CYCLE, and Lomb-Scargle, to determine the p-value, Benjamini-Hochberg q-value (BH.Q value), period, phase, baseline value, amplitude (AMP), and relative amplitude (rAMP). We defined the expression of an RNA as rhythmic when meta2d p<0.05.

## Results

### Core clock genes *Bmal1*, *Cry1/2*, and *Nr1d1/2* affect the expression patterns of *Per2AS* and *Per2*

**Figure 1.**
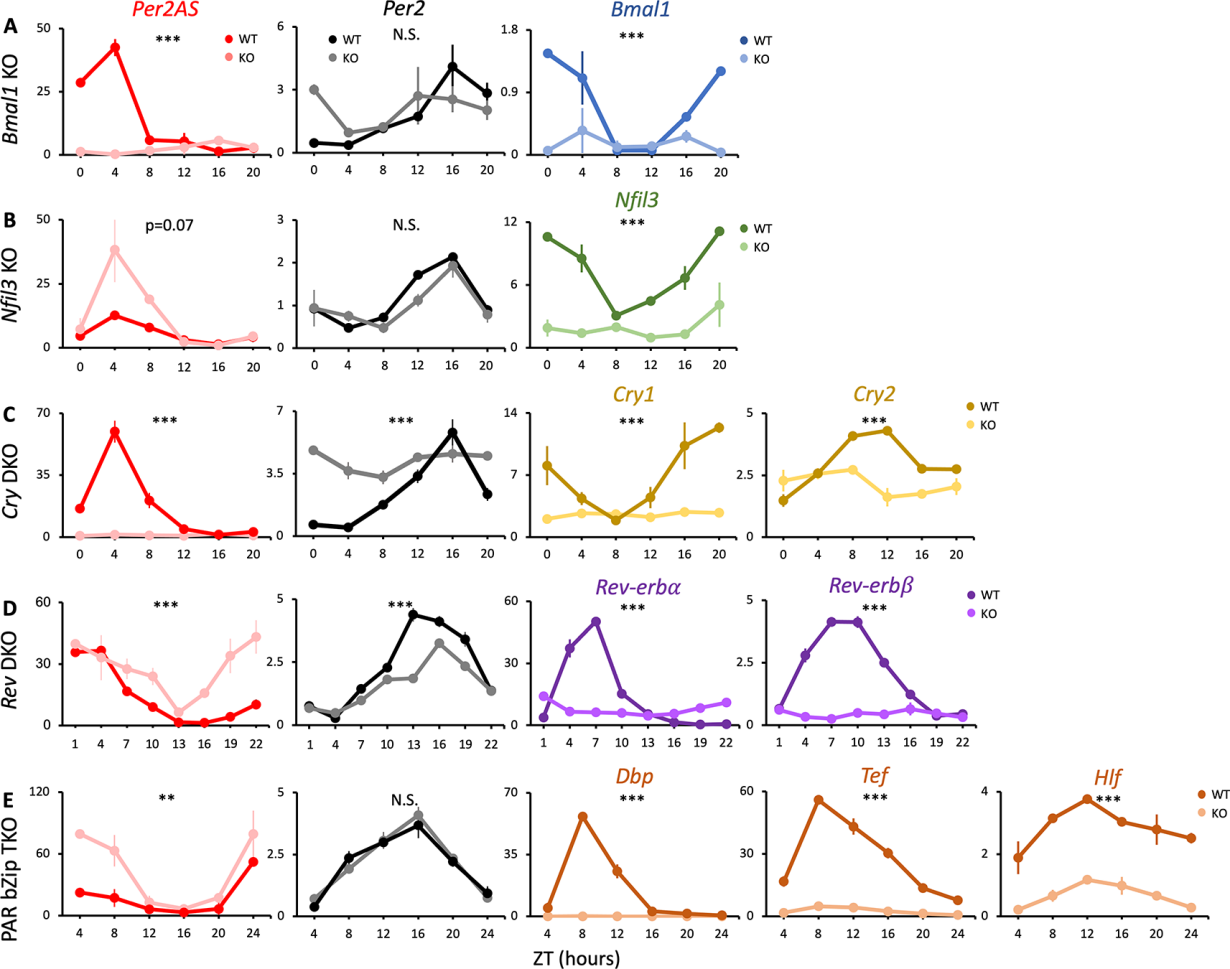
The expression patterns of *Per2AS* and *Per2* in the knockout mice of core clock genes. Mice were kept under an L:D=12:12 cycle and fed *ad libitum.* Liver RNAs were extracted every four hours in (A) *Bmal1*, (B) *Nfil3*, (C) *Cry1/2* double, and (E) *Dbp/Tef/Hlf* triple knockout mice (n=2), or every three hours in (D) *Nr1d1/2* double knockout mice (n=3). Y-axis represents strand-specific TPM. Points and error bars represent mean±SE. (**) p<0.01, (***), p<0.001, N.S. indicates no significant difference between genotypes (two-way ANOVA). Data derived from Weger *et al*., *Proc Natl Acad Sci U S A*, 2020 ^34^; Yoshitane *et al*., *Commun Biol.*, 2019 ^38^; Guan *et al*., *Science*, 2020. ^37^

To test whether any of the core clock genes have an effect on the expression of *Per2AS* and *Per2*, we analyzed the circadian transcriptomic datasets from mice livers lacking one or more “core” clock genes ( Figure 1). Circadian rhythms in each cell are driven by a cell-autonomous molecular clock, composed of a group of core clock genes that form a network of transcriptional–translational feedback loops (TTFLs) ^42^ and regulate daily rhythms in biochemistry, physiology, and behavior. In the first loop, transcription activators BMAL1 (gene name: *Arntl*) and CLOCK form a heterodimeric complex and bind to the E-box sequence in the promoter regions to regulate the rhythmic expression of their target genes, including *Per1-3* and *Cryptochrome* ( *Cry)1-2.* High levels of PER and CRY proteins form a protein complex in the cytoplasm, then translocate back to the nucleus to inhibit its own transcription by interacting with CLOCK/BMAL1. This cycle takes approximately 24 hours, and this is the molecular basis of generating circadian rhythms. The secondary feedback loop is comprised of the transcriptional repressors REV-ERBs (gene name: *Nr1d*) and the activator RORs that are regulated by BMAL1/CLOCK. REV-ERB and ROR proteins both recognize and bind to the RORE sequence in the promoter region and compete with each other to drive the rhythmicity of the target gene expression, including *Bmal1.* The last loop consists of transcription activators of proline and acidic amino acid-rich basic leucine zipper (PAR bZip) proteins: DBP, TEF, and HLF, and the repressor NFIL3, all of which target genes containing D-box element within their promoters, including *Rev-erbs, Rors*, and *Pers*. ^42^

Removal of *Bmal1* from the TTFL results in arrhythmic locomotor activity and disrupts circadian expression of hepatic core clock genes. ^43^^,^^44^ Similarly, removal of both *Cry1* and *2* also results in the loss of the rhythm in locomotor activities and core clock gene expression. ^45^^,^^46^ In both *Bmal1* knock-out (KO) and *Cry1/2* double knock-out (DKO) animals, our analysis demonstrated that the rhythmicity of *Per2* was abolished but expression was maintained at intermediate levels, as was reported previously ^34^^,^^47^^,^^48^ ( Figure 1A, C, Table S1 ^85^). Whereas the expression of *Per2AS* was markedly low and arrhythmic, indicating that *Bmal1* and *Cry1/2* promote the expression of *Per2AS* ( Figure 1A, C, Table S1 ^85^).

In contrast, the removal of *Nfil3* or PAR bZip genes ( *Dbp/Tef/Hlf)* did not change the expression pattern of *Per2* ( Figure 1B, E). This is in line with previous findings that the rhythmic expression of core clock genes was nearly identical between wild-type (WT) and *Nfil3* or PAR bZip-deficient mice in liver. ^38^^,^^49^ Interestingly, however, the amplitude of *Per2AS* increased in both *Nfil3* KO *and Dbp/Tef/Hlf* triple knock-out (TKO) mice, indicating that the NFIL3 and PAR bZip proteins repress the expression of *Per2AS* without affecting *Per2* ( Figure 1B, E, Table S1 ^85^).

Removal of both *Nr1d1* and *Nr1d2* in the SCN did not disrupt the circadian locomotor activity, but significantly shortened the free-running period. ^50^ Also, Nr1d1/2 DKO mice showed interfered circadian expression of many hepatic core clock genes, including dampening the rhythm of *Per2*. ^51^ Consistent with this, the amplitude of *Per2* expression was decreased in *Nr1d1/2* DKO mice while the relative amplitude of *Per2AS* expression was increased ( Figure 1D, Table S1 ^85^). These data suggest that *Nr1d1/2* have an opposing effect on *Per2* and *Per2AS*, activating *Per2* while repressing *Per2AS.* We confirmed the genotypes of each dataset by checking the mRNA levels of the knock-out genes, all of which were significantly decreased ( Figures 1, 3, 4). We also provided the mRNA levels of all the other core clock genes (Table S2). ^86^

### 24-hr fasting decreased the amplitude of the rhythmic expression of *Per2*, but not that of *Per2AS*

We next tested the effect of environmental perturbation on the expression patterns of *Per2AS* and *Per2.* In mammals, light is the most potent environmental cue that entrains the circadian clock through the suprachiasmatic nucleus (SCN) of the hypothalamus. However, food intake also serves as a strong ‘Zeitgeber’ (time giver) to entrain the circadian clock of peripheral organs in an SCN-independent manner. ^52^^–^^55^ Previous studies have demonstrated that fasting directly affects a large number of physiological parameters, such as body temperature, ^56^^,^^57^ body weight, ^58^^,^^59^ hormone levels, ^60^^,^^61^ and hepatic glucose levels, ^62^^,^^63^ in addition to the expression patterns of the core clock genes in mouse liver.

**Figure 2.**
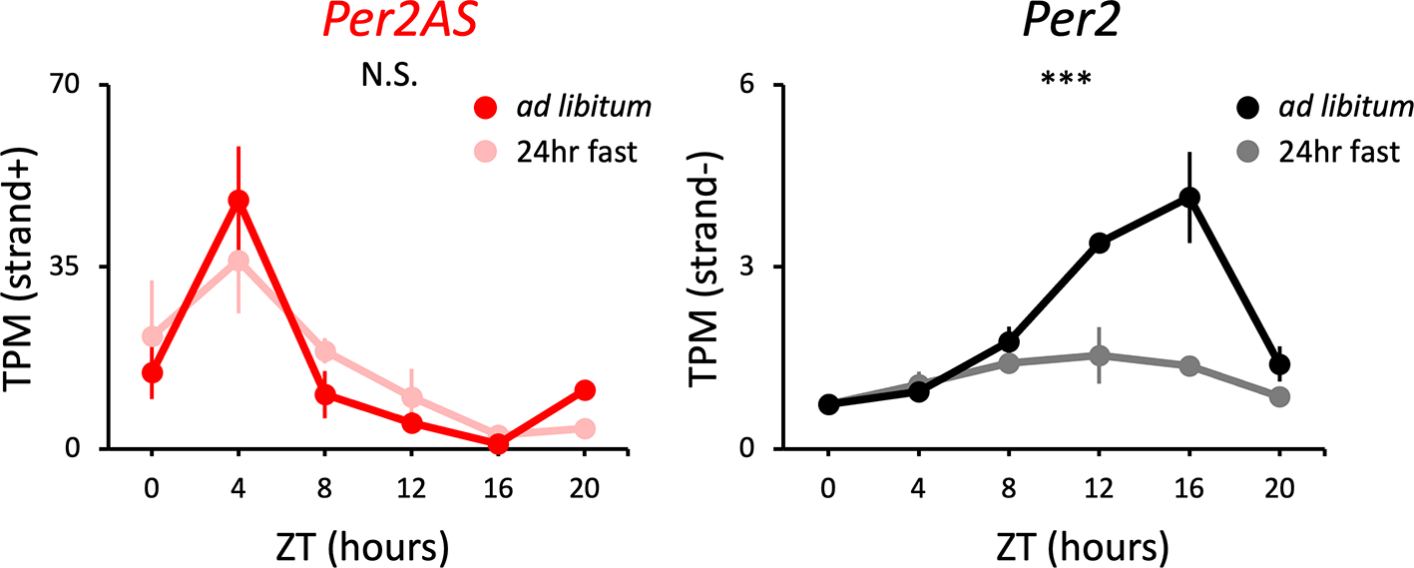
The expression patterns of *Per2AS* and *Per2* under *ad libitum* or 24-hr fasting conditions. Mice were kept under an L:D=12:12 cycle and either fed *ad libitum* or food was removed 24 hours prior to tissue sampling for the fasting group of mice. Liver RNAs were extracted every four hours. Points and error bars represent mean±SE (n=3). (***) p<0.001, N.S. indicates no significant difference between feeding conditions (two-way ANOVA). Data derived from Kinouchi *et al.*, *Cell Rep.*, 2018. ^35^

In particular, the expression levels of BMAL1-target genes, including *Per2*, are lower in the liver of fasting mice. ^35^^,^^64^^–^^68^ In line with this, the amplitude of *Per2* expression was considerably lower in the fasted mice ( Figure 2, Table S1 ^85^) whose liver samples were collected after 24 hours of fasting for each time point, compared to the mice fed under the *ad libitum (ad lib)* condition. In contrast, the expression patterns of *Per2AS* show little or no difference between the *ad lib* fed and 24-hr fasting conditions ( Figure 2, Table S1 ^85^). These data indicate that the 24-hr fasting alters the expression pattern of *Per2*, but not that of *Per2AS.*

### *Bmal1* and *Cry1/2* regulate *Per2AS* expression even under the time-restricted feeding condition

**Figure 3.**
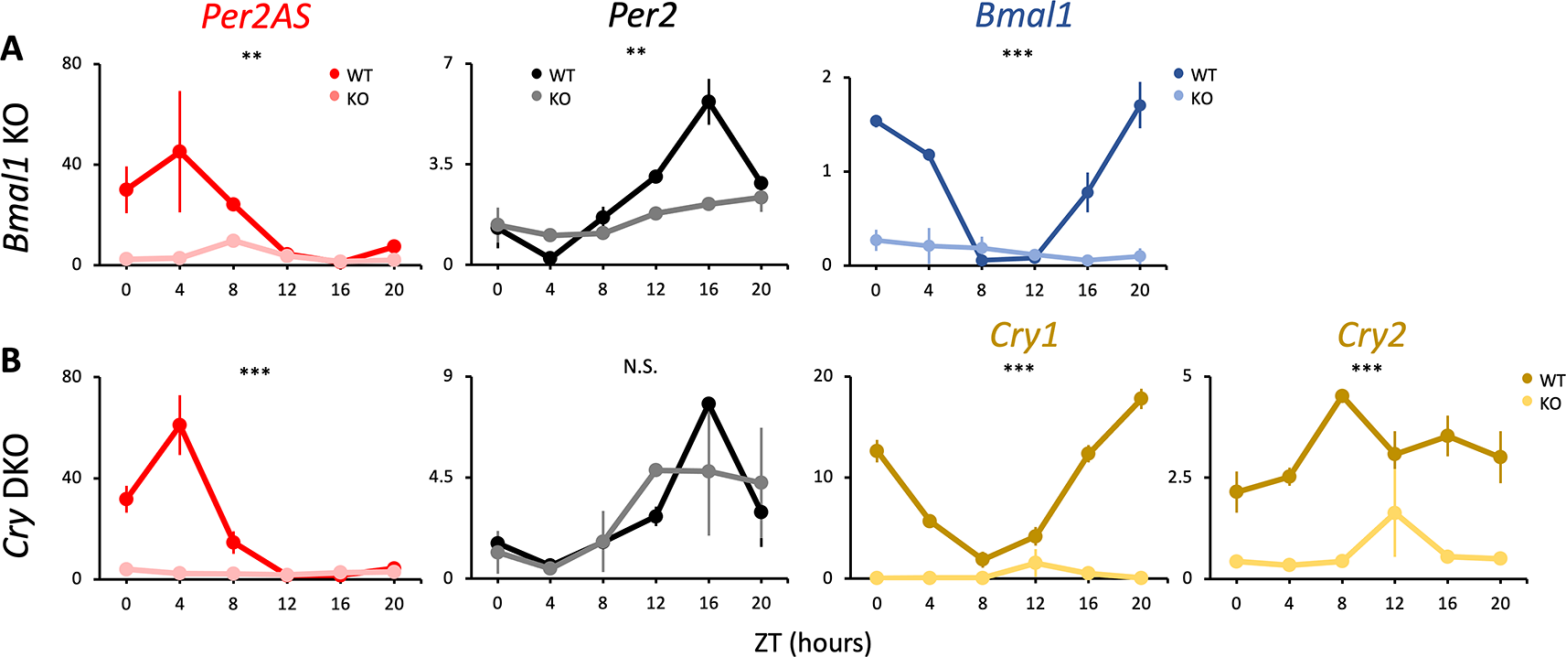
The expression patterns of *Per2AS* and *Per2* with time-restricted feeding (TRF). Mice were kept under an L:D=12:12 cycle and access to food were restricted between ZT12 to ZT24. Liver RNAs were extracted every four hours in (A) *Bmal1* and (B) *Cry1/2* double knockout mice. Y-axis represents strand-specific TPM. Points and error bars represent mean±SE (n=2). (**) p<0.01, (***) p<0.001, N.S. indicates no significant difference between genotypes (two-way ANOVA). Data derived from Weger *et al*., *Proc Natl Acad Sci U S A*, 2020. ^34^

Time-restricted feeding (TRF) is a form of intermittent fasting in which food consumption is restricted to a certain time window of the day. The TRF regimen can protect mice from excessive body weight gain and liver damage depending on the timing of the food availability, and also improve metabolic and physiological rhythms when food access is restricted to the active phase. ^69^^–^^72^ Here, we also looked at the differences in *Per2AS* and *Per2* expression patterns when food access was restricted to nighttime (i.e., the active phase of mice) to understand whether core clock genes still have the same effect on the expression of *Per2AS* and *Per2.* Similar to what was observed with the *ad lib* feeding condition ( Figure 1A, C), the expression of *Per2AS* was very low and arrhythmic in *Bmal1* KO and *Cry1/2* DKO animals experiencing TRF compared to WT ( Figure 3, Table S1 ^85^). The expression of *Per2* was also arrhythmic but still maintained intermediate levels both in *Bmal1* KO and *Cry1/2* DKO animals even under TRF ( Figure 3, Table S1 ^85^). Compared to *ad lib* feeding, TRF did not affect the expression patterns of *Per2AS* and *Per2*, showing that the core clock gene KO is the primary factor to alter the expressions of *Per2AS* and *Per2.* Additionally, these findings further support that BMAL1 and CRY are crucial transcription factors to promote *Per2AS* expression, regardless of the feeding patterns.

### Core clock genes *Bmal1*, *Cry1/2*, and *Nr1d1/*2 knockout abolished the expression of *Per2AS* and dampen the rhythm of *Per2* when feeding with HFD

In addition to the timing of diet, the composition of the diet, such as high-fat or ketogenic, also affects circadian rhythms and alters core clock gene expression. ^69^^,^^73^^–^^76^ To understand whether different diet compositions affect the expression of *Per2AS* and *Per2* differently, we also examined the expression levels of *Per2AS* and *Per2* when mice were fed with a 60% high-fat diet (HFD) either *ad lib* or TRF during the active phase (ZT13-22 or 23). ^36^

**Figure 4.**
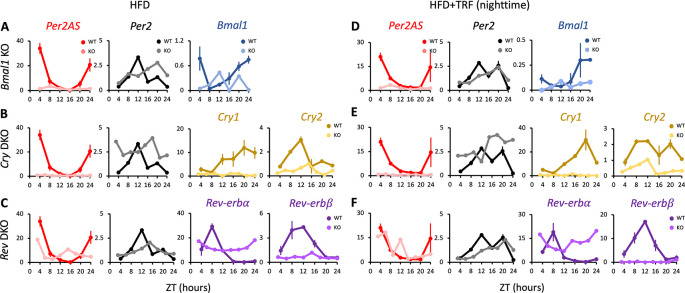
The expression patterns of *Per2AS* and *Per2* with high-fat diet (HFD). Mice were kept under an L:D=12:12 cycle and fed with a 60% high-fat diet either *ad lib* (A-C) or with TRF (D-F) condition. Liver RNAs were extracted every four hours in wild type and (A) *Bmal1* knockout mice, or every three hours in (B) *Cry1/2* double and (C) *Nr1d1/2* double knockout mice. Y-axis represents strand-specific TPM. The same WT mice were used in each group. Points and error bars represent mean±SE (n=2). Two-way ANOVA analysis was not performed for this dataset, because the KO samples consisted of a single biological replicate. Data derived from Chaix *et al*., *Cell Metab.*, 2019. ^36^

The expression of *Per2* was arrhythmic in WT fed with HFD under the *ad lib* condition ( Figure 4A, Table S1 ^85^), as was reported previously. ^74^ However, it was rhythmic under the TRF condition ( Figure 4D, Table S1 ^85^), supporting the idea that TRF restores peripheral oscillations of core clock gene expressions. ^77^^,^^78^ Similar to the results observed with a regular chow diet under the *ad lib* condition ( Figure 1), *Per2* expression was arrhythmic but maintained intermediate expression in both *ad lib* and TRF conditions in *Bmal1* KO and *Cry1/2* DKO ( Figure 4, Table S1 ^85^), but not in *Nr1d1/2* DKO mice with HFD ( Figure 4C, F, Table S1 ^85^). In contrast, *Per2AS* levels were low and arrhythmic in both *ad lib* and TRF conditions in *Bmal1* KO and *Cry1/2* DKO mice compared to WT mice with HFD ( Figure 4A- B, D- E), similar to what was observed in regular chow mice under both *ad lib* and TRF conditions ( Figure 1A, C; Figure 3). This further supports the idea that *Per2AS* expression is regulated by *Bmal1* and *Cry1/2*, regardless of the composition of the diet. On the other hand, *Per2AS* expression was arrhythmic in *Nr1d1/2* DKO mice with HFD in contrast to the regular chow diet under *ad lib* condition ( Figure 1D), suggesting that the effect of *Nr1d1/2* on *Per2AS* is different between regular chow and HFD ( Figure 4C, F; Table S1 ^85^).

## Discussion

In this study, we focused on the circadian antisense lncRNA, *Per2AS*, and asked whether the rhythmic expression of *Per2AS* is regulated independently by its own promoter like many other circadian mRNAs, or transcriptional interference driven by the antiphasic expression of its sense-strand gene *Per2.* By using circadian transcriptomic datasets from mouse liver, in which the molecular clock machinery was genetically and environmentally disrupted, we examined how these perturbations affect the expression patterns of *Per2AS* and *Per2.* Our data demonstrated that the expression of *Per2AS* can be altered by both genetic and environmental perturbations of the circadian clock.

We were able to test the effect of *Bmal1, Nfil3, Cry1/2, Nr1d1/2, and Dbp/Tef/Hlf*, but not that of *Rora/b/c* as the circadian transcriptome dataset for *Rora/c* KO mouse liver is only available with a microarray platform and this does not allow us to quantify *Per2AS* levels. ^79^ We also tested the effect of fasting, TRF, and an HFD on the expression patterns of *Per2AS* and *Per2.* Even though the expression of many core clock genes, including *Per2*, is affected by fasting ( Figure 2) or HFD ( Figure 4), ^35^^,^^36^^,^^64^^–^^68^ *Per2AS* appears to be less sensitive to these changes and its expression remained rhythmic under fasting, TRF, or HFD conditions ( Figures 2- 4). These data suggest that the timing of food intake, the composition of the diet, and fasting are not the primary factor that regulates the rhythmic expression of *Per2AS.* By contrast, the effect of genetic perturbation within the circadian rhythm system is stronger than environmental perturbation on the expression *Per2AS.*

We tested our hypotheses for *Per2AS* transcription regulation by comparing the changes in the expression pattern between *Per2AS* and *Per2.* We found that, in some cases, the *Per2AS* expression pattern was altered even when that of *Per2* was unaltered ( Figure 1B, E). We also found, in other cases, that the *Per2AS* expression pattern was unaltered even when that of *Per2* was altered ( Figure 2). These data strongly support the independent hypothesis that the expression of *Per2AS* is regulated by its own promoter, and its transcription is independent from that of *Per2.* Indeed, the expression of many lncRNAs is controlled by their promoter or other DNA elements, such as enhancers. ^30^^,^^80^ The majority of lncRNAs also contain highly conserved core promoter sequences and can be regulated by different transcription factors. ^81^^,^^82^ In addition, the promoters of lncRNAs are evolutionarily conserved as much as that of mRNAs at least between humans and mice, even though their nucleotide sequences are less conserved than mRNAs. These data support the significance of promoter sequences in regulating lncRNA expression patterns. ^83^ Our data also indicate that BMAL1 and CRY1/2 are the activators, and NFIL3 and PAR bZip proteins are the repressors of *Per2AS* ( Figure 1, 3). However, the removal of one particular core clock gene may alter the expression of other core clock genes or their downstream genes. ^34^^,^^84^ Thus, we cannot eliminate the possibility that the *Per2AS* expression is indirectly impacted by the change of the core clock gene circuit.

Although these data strongly support the independent hypothesis, we cannot completely reject the alternative hypothesis that *Per2AS* expression is regulated by transcriptional interference from *Per2*, since there are some instances where the changes in the expression pattern of *Per2AS* and *Per2* can still be explained by the transcriptional interference hypothesis. For example, in *Bmal1* KO and *Cry1/2* DKO animals under any dietary conditions (i.e., *ad lib*, TRF, and HFD feeding), the *Per2AS* expression was completely abolished, and the *Per2* expression pattern was also arrhythmic but stayed at the intermediate levels ( Figures 1A, C, 3, 4D, E, Table S1 ^85^). It is possible that the constant *Per2* expression prevents the transcription of *Per2AS* on the other strand, leading to the decreased and arhythmic *Per2AS* expression. Additionally, in *Nr1d1/2* DKO mice, *Per2AS* expression increased and its rhythmicity became more robust, while *Per2* expression decreased and its rhythmicity was dampened ( Figure 1D). This could be due to decreased *Per2* transcription leading to the increased *Per2AS* transcription on the other strand. Therefore, the two alternative hypotheses can both be viable and coordinated together to regulate the rhythmic transcription of *Per2AS.* At the same time, these data can also be explained solely by the independent hypothesis, and additional experimental evidence will be required to distinguish these possibilities. For example, it would be helpful to understand whether these core clock proteins are indeed recruited to the *Per2AS* promoter or enhancer sequences. We can also modify the transcription of *Per2* directly and test whether this would lead to changes in *Per2AS* expression patterns.

Regardless, these results help us better understand not only how the transcription of *Per2AS* is regulated, but also how *Per2AS* is wired with other core clock proteins in transcriptional-translational feedback loops to regulate circadian rhythms because the rhythmic transcription of *Per2AS* is important for its functions in regulating circadian rhythms. More broadly, our results also help us understand the transcription regulation mechanism of antisense lncRNAs.

## Data Availability

NCBI GEO: Temporal profiles of gene expression in *Cry1/2* KO, *Bmal1* KO under night restricted feeding and ad libitum feeding regimen. Accession number: GSE135898, https://www.ncbi.nlm.nih.gov/geo/query/acc.cgi?acc=GSE135898. NCBI GEO: Temporal profiles of hepatic gene expression in PAR bZip triple knockout mice. Accession number: GSE135875, https://www.ncbi.nlm.nih.gov/geo/query/acc.cgi?acc=GSE135875. NCBI GEO: Fasting Imparts a Switch to Alternative Circadian Transcriptional Pathways in Liver and Muscle. Accession number: GSE107787, https://www.ncbi.nlm.nih.gov/geo/query/acc.cgi?acc=GSE107787. NCBI GEO: Hepatic transcriptome by Next Generation Sequencing of WT and clock mutant mice fed a HFD ad libitum or time-restricted feeding. Accession number: GSE102072, https://www.ncbi.nlm.nih.gov/geo/query/acc.cgi?acc=GSE102072. NCBI GEO: The Hepatocyte Clock and Feeding Interdependently Control Chrono-Homeostasis of Multiple Liver Cell Types (RNA-seq). Accession number: GSE143524, https://www.ncbi.nlm.nih.gov/geo/query/acc.cgi?acc=GSE143524. NCBI BioProject: Transcriptome of mouse liver in *Per2::Luc* KI and *E4bp4* KO/ *Per2::Luc* KI mice. Accession number: PRJDB7789, https://www.ncbi.nlm.nih.gov/bioproject?term=PRJDB7789&cmd=DetailsSearch. figshare: Table_S1.xlsx. https://doi.org/10.6084/m9.figshare.21067537.v1. ^85^ figshare: Table_S2.xlsx. https://doi.org/10.6084/m9.figshare.21375783.v1. ^86^ This project contains the following underlying data:
‐
Table S1.xlsx (Metacycle analysis to determine whether the gene expression is rhythmic) ‐
Table S2.xlsx (RNA expression levels (strand-specific TPM) of the core clock genes) Data are available under the terms of the Creative Commons Attribution 4.0 International license (CC-BY 4.0).
