# Supplementary material for: Genetic and environmental perturbations alter the rhythmic expression pattern of a circadian long non-coding RNA, Per2AS, in mouse liver
Source: F1000Res. 2022 Oct 31;11:1073. Originally published 2022 Sep 20. [Version 2] doi: 10.12688/f1000research.125628.2 (PMC9551389; doi:10.12688/f1000research.125628.2)
Supplement: Peer review history

# Peer review: Reviewer response for version 1

**DOI:** 10.5256/f1000research.137958.r151597

**Published:** 2022-10-10

**Authors:** Yao Cai

**Affiliations:** 1 Department of Entomology and Nematology, College of Agricultural and Environmental Sciences, University of California Davis, Davis, CA, USA

Miao *et al.* investigated how genetic and environmental manipulations influence the expression of *Per2AS*, a circadian long non-coding RNA. Despite the identification of *Per2AS* in several circadian transcriptome studies and publication of a functional studies (Mosig *et al*., 2021, G&D), how *Per2AS* transcription is regulated remains a major gap in knowledge. A lncRNA transcription can be regulated via transcriptional interference. Alternatively, the promoter of a lncRNA is regulated independent of its sense RNA transcript. To gain insights on the transcriptional regulation of *Per2AS*, the authors analyzed the expression pattern of *Per2AS* and *Per2 *in response to genetic and/or environmental manipulation from publicly available RNA-seq database. *Per2AS *expression is altered in most core clock gene mutant mice. Whereas *Per2AS* expression is less sensitive to environmental manipulations including fasting, time-restricted feeding and high-fat diet. They authors suggest both models regarding transcription of the *Per2AS* lncRNA may be true. (1) In some conditions (e.g. *Cry1/2 *double knock-out) where *Per2* expression is in intermediate level throughout the day, *Per2AS* level remains in a trough level, supporting the transcriptional interference hypothesis. (2) When *Per2* expression remains in the trough level under 24-hour fasting condition, *Per2AS* level is unaltered, favoring the independent regulation hypothesis. Taken together, this manuscript provides new insights on the transcriptional regulation of *Per2AS*. Below are some minor suggestions for the authors to improve the manuscript.

1. Clarify what transcriptional interference (TI) means in the introduction section. Is there a specific TI mechanism you refer to, such as promoter competition, RNAPII collision? I believe this helps readers who are unfamiliar with TI to understand the TI hypothesis.
2. Transcripts of *Per2AS* seems to be a few fold higher than *Per2* in any given conditions (e.g., Figure 1A). Is this relevant to the two hypotheses? Can the authors comment on this phenomenon?
3. Can the authors speculate whether *Per2AS* is more sensitive to signals from the central clock than the peripheral liver clock? It is interesting that *Per2AS* is unaltered under 24-hour fasting condition (Figure 2), despite the dampening of *Per2* transcripts. Maybe analyzing *Per2AS* level in the liver of SCN lesioned mice or mice kept under constant light will be informative.
4. To date, there are no data that can solidly differentiate whether BMAL1 and CRY1/2 activates or depresses *Per2AS* transcription. For this reason, I think the word “promote” is better than “activate” when describing upregulation of *Per2AS* expression (e.g., the sentence that cites Figure 1A, C, Table S1). This will include both possibilities.
5. I have difficulty understanding the first sentence of the last paragraph in the Discussion section. What do the authors mean by “interacts with other core clock proteins”? To my understanding, this manuscript investigates how *Per2AS* transcription is influenced upon genetic manipulation on core clock genes, instead of how *Per2AS* regulates core clock proteins.

Is the work clearly and accurately presented and does it cite the current literature?

Yes

If applicable, is the statistical analysis and its interpretation appropriate?

Yes

Are all the source data underlying the results available to ensure full reproducibility?

Yes

Is the study design appropriate and is the work technically sound?

Yes

Are the conclusions drawn adequately supported by the results?

Yes

Are sufficient details of methods and analysis provided to allow replication by others?

Yes

Reviewer Expertise:

Circadian clock, phosphorylation, Drosophila genetics

I confirm that I have read this submission and believe that I have an appropriate level of expertise to confirm that it is of an acceptable scientific standard.

# Response

**Published:** 2022-10-26

**Authors:** Shihoko Kojima

1. We agree that the explanation of transcriptional interference (TI) will help readers better understand our hypothesis. We supplemented this information, and it now reads: “Our first hypothesis is that the rhythmic *Per2AS* transcription is regulated by rhythmic antiphasic transcription of *Per2* by means of transcriptional interference, in which the transcription process on one strand suppresses the transcription process of the other strand. ^27-29^” We currently do not have any specific mechanism in mind and did not mention any.

 2. The reviewer is correct that the strand-specific TPM (transcripts per million) for *Per2AS* is generally higher than that of *Per2*. This is because we had to separately calculate the strand-specific TPM on each strand. Since the total number of raw read counts is different in each strand, the expression level of *Per2* and *Per2AS* cannot be directly compared with each other. This is not relevant to the two hypotheses.

 3. We have shown that *Per2AS* is sensitive to the local liver clock as well as environmental perturbation triggered by changes in diet (Figs 1-4). It is unclear whether *Per2AS* is sensitive to the central clock (i.e., SCN). To our knowledge, there is no circadian transcriptome dataset in liver from SCN-lesioned mice.

 4. We agree with the reviewer that the word “promote” is more accurate than “activate”. We have now changed these, as suggested. 1) “Whereas the expression of *Per2AS* was markedly low and arrhythmic, indicating that *Bmal1* and *Cry1/2 *promote the expression of *Per2AS* (Figure 1A, C, Table S1 84).” 2) “Additionally, these findings further support that BMAL1 and CRY are crucial transcription factors to promote *Per2AS* expression, regardless of the feeding patterns. ”

 5. We apologize for the confusion. We revised this sentence, which now reads:

 “Regardless, these results help us better understand not only how the transcription of *Per2AS* is regulated, but also how *Per2AS* is wired with other core clock proteins in transcriptional-translational feedback loops to regulate circadian rhythms because the rhythmic transcription of *Per2AS* is important for its functions in regulating circadian rhythms.”

# Peer review: Reviewer response for version 1

**DOI:** 10.5256/f1000research.137958.r150976

**Published:** 2022-09-21

**Authors:** Hikari Yoshitane

**Affiliations:** 1 Department of Biological Sciences, School of Science, The University of Tokyo, Tokyo, Japan; 2 Circadian Clock Project, Tokyo Metropolitan Institute of Medical Science, Tokyo, Japan

Dr. Shihoko Kojima and their colleagues focused on the molecular basis of the rhythmic expression pattern of a circadian long non-coding RNA, PerAS, in the mouse liver. PerAS gene is located in the genomic locus of one of the core clock gene, Per2, and is transcribed as an antisense strand RNA. Intriguingly, PerAS shows a clear circadian rhythm of its RNA expression in the mouse liver, and the rhythmic pattern is almost anti-phasic to that of Per2 mRNA. Many chronobiologists, including me, predict that these genes probably repress each other's transcription, resulting in their anti-phasic RNA rhythms. In this study, however, the authors clearly demonstrated that Per2AS expression and Per2 mRNA expression are independently controlled by unknown mechanisms that will be revealed in the near future. They obtained a series of RNA-Seq datasets in this field to analyze RNA expression patterns of Per2AS in the mouse livers that lack core clock genes or under altered food conditions. The results are clear and the conclusion is so simple, and I strongly encourage to Approve this research in F1000Research paper with the following minor modifications.

1. The authors cite Cho *et al.*, (2012) in which conditional DKO mice of Nr1d1/2 (REV-ERBs) genes did not show clear circadian rhythms in the locomotor activity. However, this arrhythmic phenotype was probably due to the severe unhealthiness of the mutant mice, because canonical DKO mice are lethal. In Adlanmerini *et al.*, (2021) ^1^ (from the same Dr. Lazar Lab), they showed clear locomotor rhythms with significant short period in the SCN-specific DKO of REV-ERBs. Therefore, REV-ERBs are not essential genes for the circadian oscillation. This will be informative for the readers to understand the results in this paper.
2. The authors re-analyzed a series of RNA-Seq datasets in this field to analyze RNA expression patterns of Per2AS in the mouse livers. Since the bioinformatic methods for analyzing RNA-Seq data are advancing day by day and the obtained results will change depending on the method, the results analyzed by the same authors using the same method are very useful for many chronobiologists. For example in Figure 1, they showed only expression patterns of Per2AS, Per2, and the deleted genes in a series of KO mice, but I suggest them to show expression patterns of core clock genes in all the case probably in supplemental figures; especially E-box genes (Dbp and Rev-erb) expression in Bmal1-KO and Cry-DKO mice and RRE genes (Bmal1 and E4bp4) expression in Rev-erb-DKO. These results will support the quality of their bioinformatic method in this paper and also will show the quality of the raw data in the previous studies.

Minor:

1. I find it easier to compare the gene expression patterns if panels are displayed in the same size even between different figures. For example, panels in figure 2 is too big and figure 4 is too small.

Is the work clearly and accurately presented and does it cite the current literature?

Yes

If applicable, is the statistical analysis and its interpretation appropriate?

Yes

Are all the source data underlying the results available to ensure full reproducibility?

Yes

Is the study design appropriate and is the work technically sound?

Yes

Are the conclusions drawn adequately supported by the results?

Yes

Are sufficient details of methods and analysis provided to allow replication by others?

Yes

Reviewer Expertise:

Circadian clock, post-translational modifications, phosphorylation, LC-MS/MS

I confirm that I have read this submission and believe that I have an appropriate level of expertise to confirm that it is of an acceptable scientific standard.

## References

[1] : REV-ERB nuclear receptors in the suprachiasmatic nucleus control circadian period and restrict diet-induced obesity. *Sci Adv* .2021;7(44) : 10.1126/sciadv.abh2007 eabh2007 10.1126/sciadv.abh2007 34705514PMC8550249

# Response

**Published:** 2022-10-26

**Authors:** Shihoko Kojima

1. We thank the reviewer for the suggestion. We have added the paper that the reviewer mentioned, and edited the last paragraph of the section “Core clock genes *Bmal1*, *Cry1/2*, and *Nr1d1/2* affect the expression patterns of *Per2AS* and *Per2*”.

 2. We agree with the reviewer. We have now provided an additional extended data file as Table S2, which includes RNA expression levels (i.e., strand-specific TPM) of the core clock genes as well as the rhythmicity analysis of the expression patterns with MetaCycle (doi: 10.6084/m9.figshare.21375783). We also mentioned this extended data by the end of the session “Core clock genes *Bmal1*, *Cry1/2*, and *Nr1d1/2 *affect the expression patterns of *Per2AS *and *Per2*”.

 3. Unfortunately, we, the authors, have no control over the size of the figures and are not able to change their sizes.
